# Reprogramming adoptive cell therapy for osteosarcoma: engineering, vaccination, and tumor microenvironment remodeling

**DOI:** 10.3389/fimmu.2026.1865742

**Published:** 2026-06-05

**Authors:** Shiguo Zuo, Na Cheng, Zhiying Hou, Yilong Yang, Quanliang Tian, Yisheng Xu

**Affiliations:** 1 Department of Spine Surgery, Qianxinan Autonomous Prefecture Hospital of Traditional Chinese Medicine, Xingyi, Guizhou, China; 2 Department of Psychology, Xijing Hospital 986 Hospital, Fourth Military Medical University, Xi’an, Shaanxi, China; 3 Department of Orthopedics, Qianxinan Autonomous Prefecture Hospital of Traditional Chinese Medicine, Xingyi, Guizhou, China; 4 Department of Orthopedics, Guangdong Provincial Hospital of TraditionalChinese Medicine, Guangzhou, Guangdong, China

**Keywords:** adoptive cell therapy, antigen heterogeneity, CAR-T cells, cellular engineering, immunotherapy, osteosarcoma, tumor microenvironment, vaccination

## Abstract

Osteosarcoma remains a major treatment challenge, especially for patients with recurrent, refractory or metastatic diseases. Adoptive cell therapy (ACT), including CAR-T cells, TCR engineered T cells, CAR-NK cells and macrophage based cell therapy, provides a promising strategy for redirecting immune effector cells to fight osteosarcoma. However, clinical translation has been limited by antigen heterogeneity, on-target/off-tumor toxicity, insufficient tumor trafficking, poor persistence, functional exhaustion, and the immunosuppressive tumor microenvironment. This mini review discusses emerging innovations designed to overcome these safety and efficacy barriers. First, engineering strategies such as multi-antigen recognition, logic-gated CAR systems, suicide switches, transient CAR expression, armored cytokine circuits, checkpoint-resistant designs, and chemokine receptor modification may improve precision, controllability, and durability. Second, vaccination approaches may serve as programmable amplifiers of ACT by promoting *in vivo* expansion, immune memory, antigen spreading, and local inflammatory priming. Third, tumor microenvironment remodeling through stromal modulation, vascular normalization, myeloid reprogramming, checkpoint blockade, and metabolic intervention may convert osteosarcoma into a more permissive niche for cellular therapy. Collectively, next-generation ACT for osteosarcoma will likely require modular, biomarker-guided combinations that integrate cellular engineering, vaccine-based boosting, and microenvironmental remodeling to achieve safer and more durable antitumor responses.

## 1 Introduction

Osteosarcoma is the most common primary malignant bone tumor in children, adolescents, and young adults, with a strong tendency for early hematogenous dissemination, particularly to the lungs (1, 2). In the past few decades, the standard treatment mode has not changed significantly, mainly including neoadjuvant chemotherapy, complete surgical resection and adjuvant chemotherapy. This multimodal treatment method significantly improved the survival rate of patients with localized diseases; However, the prognosis of patients with recurrent, refractory or metastatic osteosarcoma is still poor (3, 4). The limited progress in this field highlights the urgent need for treatment strategies that can overcome chemotherapy resistance, clear micrometastasis lesions and establish durable anti-tumor immune surveillance.

Immunotherapy has reshaped the therapeutic pattern of a variety of malignant tumors, but its success in osteosarcoma is still limited. Immunosuppressive checkpoint inhibitors show limited clinical activity in the unselected population of osteosarcoma patients, which may be related to the low baseline T cell infiltration level, antigen expression heterogeneity, immunosuppressive tumor microenvironment and the existing lack of anti-tumor immunity (5, 6). These limitations promote the increasing interest in adoptive cell therapy (ACT). ACT aims to redirect or enhance immune effector cells *in vitro*, and then reinfusion to patients. The ACT platform includes chimeric antigen receptor T cells (CAR-T), T cell receptor engineered T cells (TCR-T), tumor infiltrating lymphocytes, CAR natural killer cells and macrophage based cell therapy, which provides an opportunity to bypass some defects of endogenous anti-tumor immunity and deliver tumor reactive immune cells with clear functional characteristic (6, 7).

Although the prospect is promising, osteosarcoma still has several obstacles, which limit the clinical impact of ACT. Candidate targets such as HER2, GD2 and B7-H3 belong to tumor associated antigens, rather than tumor specific antigens in the strict sense, which raises concerns about the toxicity of “on target/off tumor”. In addition, the heterogeneity of antigens in primary tumors, metastatic lesions and treatment resistant clones may lead to immune escape from a single antigen target. Even if tumor recognition is achieved, the reinfusion cells must still migrate to bone or lung lesions, penetrate the dense matrix and extracellular matrix barrier, resist the influence of inhibitory cytokines and myeloid cells, and maintain the effector function under the conditions of hypoxia and adverse metabolism (8). Therefore, the next generation of osteosarcoma ACT must go beyond simple cytotoxic redirection to a safer, more durable, and more suitable for the biological characteristics of solid bone tumors. This mini review focuses on three complementary innovative directions: engineering strategies to improve the accuracy and effectiveness of ACT, vaccination methods to enhance cellular immune response, and tumor microenvironment remodeling strategies to transform osteosarcoma into a microenvironment niche more conducive to sustained ACT activity.

## 2 Engineering ACT for safer and more effective osteosarcoma targeting

### 2.1 Precision targeting and safety control

The primary condition for the successful development of osteosarcoma ACT is to identify targets that are fully expressed in tumor cells but not expressed or only expressed at low levels in important normal tissues. In this context, a variety of tumor associated antigens have been studied, including HER2, GD2 and B7-H3. HER2 targeted CAR-T cells have shown feasibility and acceptable safety in early sarcoma research, including patients with osteosarcoma, although objective remission is still limited (9, 10) (**Table 1**). GD2 is another attractive target because it is expressed in osteosarcoma and other solid tumors in children; GD2 targeted cell therapy also provides an important clinical and translational research framework for testing ACT in young patients with solid malignancies (11, 12). B7-H3, also known as *CD276*, has emerged as a particularly promising target because it is broadly expressed in pediatric solid tumors and may contribute to immune evasion, tumor invasion, and metastatic behavior (13, 14). However, these antigens are not completely osteosarcoma specific, and their expression in some normal tissues suggests the possibility of “on target/off tumor” toxicity. For osteosarcoma patients with lung metastasis, this problem is particularly important, because local inflammation in lung tissue may cause serious clinical consequences.

**Table 1:** Engineering strategies to improve safety and efficacy of adoptive cell therapy in osteosarcoma.

Engineering focus	Representative strategies	Main purpose	Relevance to osteosarcoma
Target selection	HER2-, GD2-, and B7-H3-directed ACT	Redirect immune cells toward tumor-associated antigens	Provides feasible targets, but expression is heterogeneous and not fully tumor-specific
Multi-antigen recognition	Dual CARs, tandem CARs, bispecific CARs, pooled or sequential CAR products	Reduce antigen-negative escape and broaden tumor recognition	Useful for spatially and temporally heterogeneous primary, metastatic, and relapsed lesions
Logic-gated control	synNotch circuits, AND-gate CARs, inhibitory CARs	Improve discrimination between malignant and normal tissues	May lower on-target/off-tumor toxicity when antigens are shared with selected normal tissues
Safety switches	Inducible caspase-9, transient mRNA CARs, small-molecule-regulated or split CAR systems	Provide external control over ACT activity and persistence	Important because ACT products are living drugs capable of expansion, migration, and prolonged persistence
Cytokine armoring	IL-7, CCL19, IL-12, and IL-15 expression	Enhance expansion, survival, immune recruitment, and local activation	May improve activity in poorly inflamed tumors, but systemic inflammation requires careful control
Checkpoint resistance	PD-1 disruption, dominant-negative PD-1, switch receptors	Protect transferred cells from inhibitory signaling	Relevant in tumors with checkpoint ligand expression and repeated antigen exposure
TGF-β resistance	Dominant-negative TGF-β receptor, TGF-β trap	Prevent suppression by TGF-β-rich solid tumor niches	May support ACT persistence and function in immunosuppressive osteosarcoma lesions
Trafficking enhancement	*CXCR1, CXCR2, CXCR4*, or *CCR2b* engineering	Improve homing toward tumor-derived chemokine gradients	Addresses poor trafficking to primary bone lesions and pulmonary metastases
Cell-product optimization	Central memory or stem-cell memory T cells; CAR-NK and CAR-macrophage platforms	Improve persistence, reduce toxicity, or remodel myeloid-rich TME	Broadens ACT design beyond short-term cytotoxicity toward durability and immune coordination

ACT, adoptive cell therapy; CAR, chimeric antigen receptor; TME, tumor microenvironment.

The spatial and temporal heterogeneity of osteosarcoma further increases the complexity of targeting accuracy. There may be differences in antigen expression among primary bone tumors, lung metastases, residual lesions after chemotherapy and recurrent samples. Therefore, ACT products targeting a single antigen may selectively clear antigen positive clones while allowing low antigen expression or antigen negative cell population expansion (15). Therefore, multi antigen recognition system has become an important engineering strategy. Dual car and bispecific CAR designs can recognize more than one tumor associated antigen, which may reduce antigen negative escape. Tandem car integrates two antigen binding domains into one receptor, while hybrid or sequential CAR-T strategies may expand immune pressure in heterogeneous tumor cell populations (16). More complex logic gating platform, including synNotch circuits and AND-gate CAR systems, can limit the complete activation of T cells to tumor cells expressing two kinds of antigens at the same time, thus improving the ability to distinguish between malignant tissues and normal tissues (17). On the contrary, inhibitory car can be designed to recognize antigens on healthy tissues and transmit inhibitory signals to protect normal cells from car mediated killing.

Safety control is also important, because ACT products are “living drugs” that can be expanded, migrated and retained for a long time. For osteosarcoma, because the therapeutic targets are usually tumor related rather than tumor specific, the engineering strategy of external control of cell activity may be particularly valuable. Inducible suicide switches, such as the inducible caspase-9 system, can selectively clear reinfused cells in the event of severe toxicity. Transient CAR expression through mRNA electrotransfection can reduce the risk of long-term extratumor effects, but repeated administration may be necessary to maintain the anti-tumor pressure. Other methods, such as small molecules regulating CAR, split CAR system and adjustable signal domain, also provide more opportunities for regulating cell activity after infusion (18). Overall, these strategies suggest that the future osteosarcoma ACT should not only be designed as a continuously activated cytotoxic cell, but should be constructed as a controllable immune product with adjustable specificity, intensity and duration.

### 2.2 Potency engineering and functional persistence

Only improving the targeting specificity may not be enough to produce lasting curative effect, unless ACT cells can reach the tumor site, expand after infusion, resist exhaustion, and maintain cytotoxic function in the immunosuppressive environment of osteosarcoma. ACT in solid tumors often fails because reinfusion cells show insufficient migration, limited persistence in tumors, and gradual dysfunction after repeated antigen stimulation (19, 20). In osteosarcoma, these challenges may be further aggravated by the anatomical location of primary bone lesions, the presence of mineralized matrix and dense stroma, and the frequent occurrence of lung metastases with different immune and vascular characteristics. Therefore, effectiveness engineering must not only solve the problem of how ACT cells recognize tumor cells, but also solve the problem of how ACT cells migrate, survive and function after encountering adverse tissue environment.

One method is to construct “armored” ACT products that can deliver supporting cytokines or immunomodulatory molecules at tumor sites. CAR-T cells engineered to express the cytokine IL-7 and the chemokine CCL19 may improve T-cell survival and recruit endogenous immune cells, which may transform tumors with weak inflammatory response into lesions with more immune activity (21). IL-12 armored CAR-Tcells can enhance cytotoxicity, antigen presentation and innate immune activation, but systemic IL-12 exposure may lead to significant toxicity, so local or inducible expression strategies are preferable (22). IL-15 can support the persistence of T cells and NK cells, and may be especially suitable for CAR-NK platform; CAR-NK platform has potential advantages, such as low risk of graft-versus-host disease and cytokine release syndrome, and is suitable for off-the-shelf manufacturing (23). However, the armor of cytokines must be carefully balanced, because excessive immune activation may increase systemic inflammation or aggravate tissue damage in patients with high tumor load.

Resistance to inhibitory signals is another important engineering goal. *PDCD1* knockout, dominant negative PD-1 receptors, or conversion receptors that convert inhibitory signals into activated signals, may improve the function of ACT in tumors with high PD-L1 expression (24). Similarly, the dominant negative TGF-β receptor or TGF-β capture design may protect the reinfused cells from one of the strongest immunosuppressive cytokines in solid tumors (25). Because insufficient trafficking is also a major limiting factor, chemokine receptor engineering is receiving increasing attention. ACT cells can be modified to express chemokine receptors such as *CXCR1, CXCR2, CXCR4*, or *CCR2b*, enabling them to migrate along the gradient of chemokines produced by tumor cells or stromal cells (26). In parallel, manufacturing strategies that enrich for central memory, stem-cell memory, or less differentiated T-cell phenotypes may improve proliferative capacity and persistence after infusion. Emerging platforms such as CAR-macrophages may further complement T-cell-based ACT by enhancing phagocytosis, antigen presentation, and remodeling of the myeloid-rich tumor microenvironment (27). Collectively, these approaches redefine potency as a multidimensional property involving trafficking, survival, resistance to suppression, and coordination with endogenous immunity, rather than short-term cytotoxicity alone.

## 3 Vaccination strategies as amplifiers of adoptive cell therapy

Vaccination strategy may play a unique role in promoting osteosarcoma oriented ACT. Its value does not necessarily lie in being a separate anti-tumor intervention measure, but in being a programmable amplifier for cell therapy (28, 29).One of the main limitations of ACT in solid tumors is that the reinfusion cells often fail to expand enough *in vivo*, lose the effect function after repeated antigen exposure, or fail to establish a lasting immune memory after infusion. Vaccine can enhance the persistence and function of ACT by providing controllable antigen stimulation, which may solve this problem. One attractive strategy is to use virus specific T cells, such as cytomegalovirus, Epstein Barr virus, or varicella zoster virus specific T cells, as vectors for car constructs; Subsequent exposure to viral antigens or clinically available vaccines may selectively re stimulate these car modified cells and enhance their amplification, rather than relying entirely on tumor antigen binding (30–32). This approach may be particularly suitable for osteosarcoma in children and adolescents, because reducing the need for enhanced lymphatic clearance and limiting systemic cytokine toxicity are important clinical priorities. In addition to the virus enhancement strategy, dendritic cell vaccines, tumor lysate vaccines and personalized new antigen vaccines may also supplement ACT by activating endogenous anti-tumor T cells and promoting epitope expansion (33–35). This is particularly important in osteosarcoma, because antigen expression may be different among primary tumors, lung metastases and treatment resistant clones. By extending the scope of immune recognition beyond a single CAR or TCR target, vaccination may reduce the possibility of antigen negative recurrence and help maintain immune pressure against heterogeneous tumor cell populations. Dendritic cell-based methods may be particularly useful because they can enhance antigen presentation and support the initiation of polyclonal T cell responses; The vaccine based on new antigen can provide more individualized strategies for patients with tumor carrying immunogenic mutations or structural mutations. However, the effectiveness of these methods will depend on antigen selection, vaccine platform, timing of infusion relative to ACT, and the ability of osteosarcoma microenvironment to support effective immune activation. *In situ* vaccination method further connects ACT and microenvironment remodeling. Radiotherapy, oncolytic viruses, sting or Toll like receptor agonists, and other strategies to induce the death of immunogenic cells can transform tumor lesions into endogenous vaccine sites by releasing tumor antigens, enhancing the cross presentation of dendritic cells, and inducing inflammatory chemokines that promote the recruitment of immune cells (36, 37). When these interventions are applied before or after ACT infusion, they may increase local antigen accessibility, improve migration signals, and stimulate endogenous immunity while reinfusion cells play a role. In this context, vaccination provides both antigen breadth and spatial guidance for ACT cells.

However, excessive inflammation may also increase tissue toxicity, especially in patients with lung metastasis, so the balance should be achieved through careful selection of dose, route of administration and biomarker based monitoring (38). In general, the most promising role of vaccines in osteosarcoma may be to transform ACT from a one-time cell infusion into a dynamic, enhanced and self-expanding immune platform, which can continuously exert pressure on heterogeneous and evolving tumor cell populations.

## 4 Remodeling the osteosarcoma tumor microenvironment to support ACT

### 4.1 Overcoming stromal, vascular, and trafficking barriers

There are many physical and biological obstacles in the tumor microenvironment of osteosarcoma, which can prevent ACT cells from reaching and infiltrating tumor lesions. Unlike hematological malignancies, osteosarcoma occurs in a complex bone related niche, characterized by mineralized matrix, osteoid formation, extracellular matrix deposition, abnormal blood vessels, stromal cells, and varying degrees of hypoxia (39–42). These characteristics can form a space limited structure, so that the immune effector cells are excluded from the tumor nest, or remain in the perivascular and matrix areas. The challenge is not limited to primary bone tumors. Lung metastasis is the most common site of osteosarcoma recurrence. Compared with the primary lesion, lung metastasis may have different vascular, matrix and immune characteristics, which means that ACT products must be able to migrate to tumor sites with different anatomical and biological characteristics. Therefore, the lack of infiltration in osteosarcoma should not be simply understood as delivery failure, but should be regarded as an active microenvironment barrier created by bone matrix, matrix rejection, chemokine imbalance and abnormal vascular pathways.

The mismatch of chemokines is one of the most important and possibly targeted reasons for the low migration efficiency of ACT. Reinfused T cells or NK cells may lack chemokine receptors required to follow chemokine gradients produced by osteosarcoma cells, cancer-related fibroblasts, endothelial cells, or inflammatory myeloid cell populations (43). Engineering ACT cells to express CXCR1, CXCR2, CXCR4 or CCR2b receptors may improve their homing ability to tumor derived chemokines, especially when used in combination with therapies that increase the production of local chemokines. Local radiotherapy can participate in this process by inducing inflammatory signals, increasing antigen release and promoting immune cell recruitment. Therefore, it can be used as both a cytotoxic treatment method and a microenvironment pretreatment method for ACT (44). The vascular normalization strategy may also improve the ability of immune cells to enter tumors by reducing abnormal vascular structure, hypoxia and uneven perfusion (45). At the same time, extracellular matrix remodeling, local delivery methods and regional administration may help to overcome physical rejection, especially in lesions that are large or difficult to reach by surgery (46). These strategies are unlikely to work alone; Instead, they should match each patient’s major barriers. For example, tumors with high matrix density may require matrix remodeling, while lesions with low chemokine expression may benefit more from chemokine induced therapy or receptor engineered act. Therefore, remodeling the matrix and migration barrier should be considered as an essential part of the ACT design of osteosarcoma.

### 4.2 Reprogramming immunosuppressive and metabolic niches

Even if ACT cells successfully enter osteosarcoma lesions, they will still encounter inhibitory immune and metabolic conditions, which can quickly damage their cytotoxic function. The osteosarcoma microenvironment contains tumor-associated macrophages, myeloid-derived suppressor cells, regulatory T cells, inhibitory cytokines, immune checkpoint ligands, and metabolically hostile zones marked by hypoxia, nutrient competition, lactate accumulation, and adenosine signaling (47, 48). These factors can reduce T cell receptor signal transduction, inhibit cytokine production, promote depletion, and limit the survival of reinfused cells. Myeloid cell populations are particularly important because they can support anti-tumor immunity or enhanced immune escape according to their polarization state. Tumor associated macrophages and myeloid derived suppressor cells can inhibit act through arginine depletion, reactive oxygen species, IL-10, TGF - β and immune checkpoint ligand expression. Therefore, myeloid cell reprogramming has become the core strategy to transform osteosarcoma from ACT-resistant niche to ACT-supportive environment.

**Figure 1:**
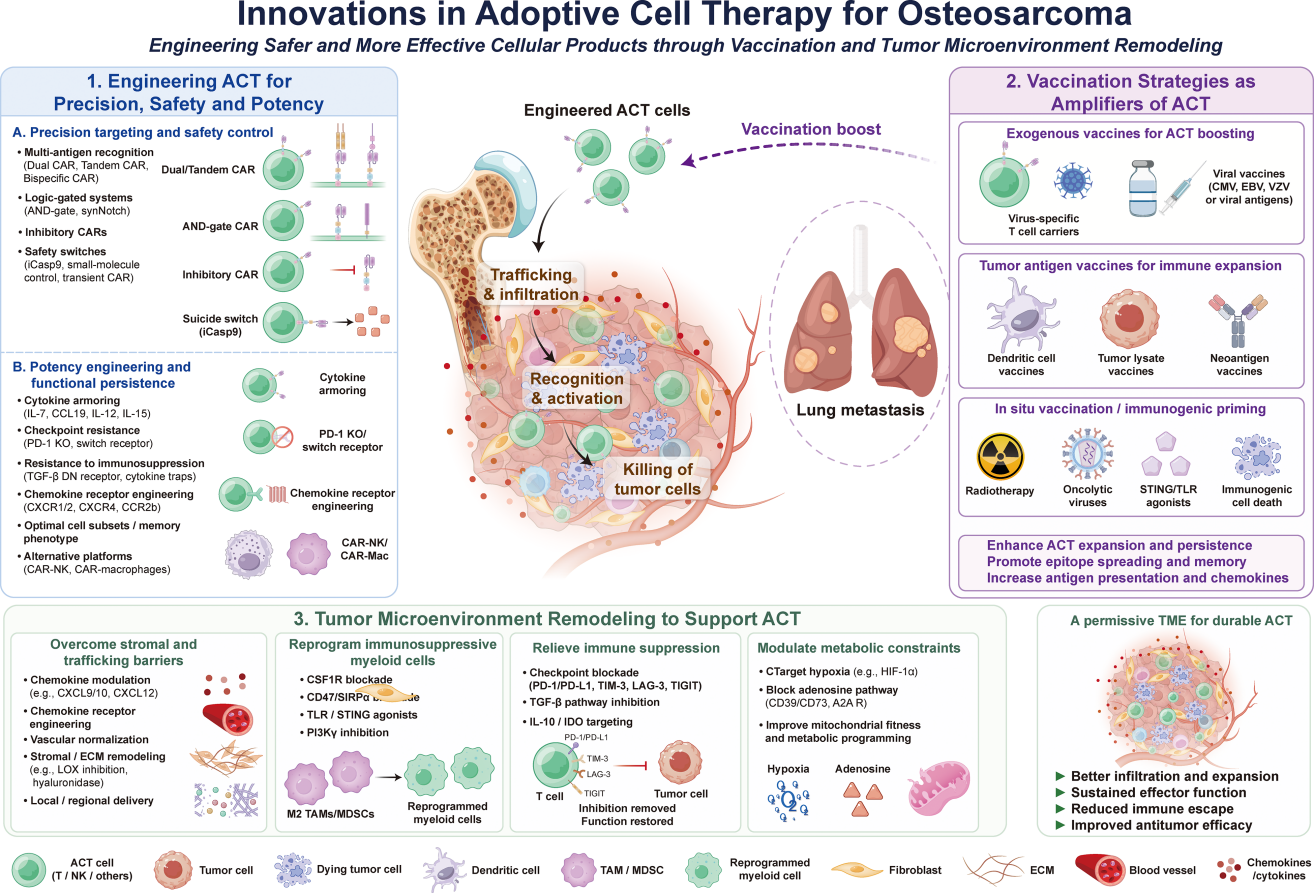
Tumor microenvironment remodeling strategies to enhance adoptive cell therapy in osteosarcoma. The osteosarcoma tumor microenvironment (TME) contains multiple barriers that limit the efficacy of adoptive cell therapy (ACT), including mineralized bone matrix, dense extracellular matrix, abnormal vasculature, chemokine mismatch, hypoxia, suppressive myeloid cells, inhibitory checkpoints, and metabolic stress. Strategies such as vascular normalization, extracellular matrix remodeling, chemokine receptor engineering, radiotherapy-induced immune priming, CSF1R blockade, CD47/SIRPα blockade, TLR/STING activation, PI3Kγ inhibition, checkpoint blockade, TGF-β pathway inhibition, adenosine-axis targeting, and metabolic fitness enhancement may improve ACT trafficking, infiltration, persistence, and effector function. Together, these approaches may convert osteosarcoma from an ACT-resistant niche into a more permissive immune microenvironment capable of supporting durable cellular antitumor responses.

A variety of methods can be used to remodel the inhibitory medullary system and metabolic network. CSF1R blockade can reduce or reprogram the macrophage population, while CD47/SIRPα blockade may enhance phagocytosis and improve antigen presentation (49). TLR or sting agonists can stimulate innate immune activation, type I interferon signal transduction, dendritic cell maturation, and inflammatory chemokines production, thus supporting both endogenous immunity and reinfusion of ACT cells. PI3Kγ inhibition may further promote macrophages from immunosuppressive phenotype to more inflammatory phenotype (50). Immune checkpoint access, including PD-1/PD-L1, TIM-3, LAG-3, and TIGIT, may also lead to act dysfunction, especially after repeated antigen exposure (51). These signals can be targeted by systemic antibodies, local delivery systems, or cellular intrinsic engineering methods, such as PD-1 knockout and receptor conversion. In addition, TGF-β, IL-10 and Ido are important soluble immunosuppressive mediators, which can be responded by dominant negative receptors, cytokine traps or combined therapy (52). Metabolic remodeling is another emerging frontier. Hypoxia responsive CAR design, A2A receptor blockade, and targeting the CD39/CD73 adenosine axis may help reinfusion cells maintain activity under hypoxia and adenosine rich conditions (53). During the preparation process, enhancing mitochondrial fitness or screening T cell subsets with stronger metabolic toughness may further improve the durability after infusion. Ultimately, TME remodeling should not be viewed as an optional adjunct to ACT. In osteosarcoma, where immune exclusion, myeloid suppression, and metabolic stress converge, durable cellular therapy will likely require simultaneous targeting of tumor cells and the ecological niche that protects them (**Figure 1**).

## 5 Discussion and future perspectives

The future development of ACT for osteosarcoma will likely require a shift from single-agent, single-target strategies toward modular, biomarker-guided, and dynamically adaptable combination therapy (54, 55). Although CAR-T, TCR-T, CAR-NK, and macrophage-based approaches have expanded the therapeutic imagination for relapsed or refractory osteosarcoma, their clinical impact will remain limited unless antigen specificity, safety, persistence, trafficking, and microenvironmental resistance are addressed together. Future trials should therefore integrate patient selection with product design. Tumor profiling should evaluate not only candidate antigens such as HER2, GD2, and B7-H3, but also spatial antigen heterogeneity, HLA-restricted antigen presentation, immune-cell infiltration, tumor-associated macrophage and myeloid-derived suppressor cell signatures, checkpoint expression, stromal density, and metabolic suppressive pathways such as hypoxia and adenosine signaling. These biomarkers could guide whether a patient is more likely to benefit from a single-target CAR, a multi-antigen logic-gated product, a vaccine-boosted ACT platform, or a combination incorporating TME remodeling.

A more explicit precision-medicine framework may further strengthen the translational relevance of ACT in osteosarcoma. Because osteosarcoma has significant interpatient and intratumoral, spatial and temporal heterogeneity, ACT selection should ideally be guided by integrated molecular and immune spectrum analysis, rather than just based on a single antigen positive (56). Multiomics methods, including bulk RNA sequencing and single-cell RNA sequencing, spatial transcriptomics, multiple immunophenotypic analysis, proteomics, and circulating tumor DNA analysis, may help to determine whether tumors are dominated by antigen heterogeneity, immune rejection, myelosuppression, antigen presentation defects, or metabolic resistance (57). For example, the high expression and uniform spatial distribution of HER2, GD2 or B7-H3 may support the corresponding single-target ACT strategy; The heterogeneous or inconsistent antigen expression between primary and metastatic lesions may be more suitable for dual target, tandem, hybrid or logic gated CAR design. Similarly, tumors rich in inhibitory macrophages, TGF - β signaling, hypoxia or adenosine metabolism may require the combination of act and TME remodeling drugs, or the use of engineering design with cell intrinsic resistance (**Table 2**) (58).

**Table 2:** Biomarker-informed precision-medicine framework for ACT selection in osteosarcoma.

Profiling layer/biomarker	What it may reveal	Potential ACT implication
HER2, GD2, B7-H3 expression	Target abundance, spatial distribution, and inter-lesion heterogeneity	Selection of HER2-, GD2-, or B7-H3-directed CAR-T/CAR-NK strategies
Multi-antigen expression patterns	Co-expression or mutually exclusive expression of candidate targets	Dual CARs, tandem CARs, pooled CAR products, or logic-gated designs
Single-cell RNA sequencing	Tumor subclones, immune-cell states, exhausted T cells, and suppressive myeloid populations	Identification of resistant clones and rational combination strategies
Spatial transcriptomics/multiplex imaging	Spatial relationships among tumor cells, immune cells, vasculature, and stromal barriers	Regional delivery, trafficking enhancement, stromal remodeling, or logic-gated ACT
HLA expression and antigen-presentation machinery	Suitability for HLA-restricted TCR-T or neoantigen-based strategies	Selection of TCR-T, vaccine-boosted ACT, or non-HLA-restricted CAR approaches
Immune phenotyping	T-cell infiltration, macrophage/MDSC abundance, and checkpoint ligand expression	Checkpoint-resistant ACT, macrophage reprogramming, or checkpoint blockade combinations
Hypoxia, adenosine, and metabolic signatures	Metabolic barriers limiting ACT persistence and cytotoxicity	A2A receptor blockade, *ENTPD1/NT5E* targeting, or metabolically optimized ACT cells
Circulating tumor DNA	Tumor burden, minimal residual disease, molecular relapse, and emerging resistant clones	Longitudinal monitoring and adaptive modification of ACT strategy

ACT, adoptive cell therapy; CAR, chimeric antigen receptor; CAR-NK, chimeric antigen receptor natural killer cell; ctDNA, circulating tumor DNA; MDSC, myeloid-derived suppressor cell; TME, tumor microenvironment.

Although highly engineered ACT products are attractive in concept, its clinical transformation in osteosarcoma must be considered from a practical perspective. Logic gated CARs, inducible cytokine circuit, anti checkpoint design and metabolic reprogramming cells may improve specificity and function, but each additional layer of engineering transformation may increase production complexity, release detection requirements, regulatory uncertainty, preparation time and cost (59, 60). These challenges are particularly relevant for osteosarcoma, because osteosarcoma is a rare disease that mainly affects children, adolescents and young adults (56). The number of patients is limited, and the rapid progress of the disease may limit the feasibility of individualized autologous cell preparation. Therefore, the most complex cell product is not always the most clinically feasible choice. More practical methods, including transient mRNA CAR expression, regional or repeated administration, off the shelf CAR-NK platform, virus-specific T-cell carriers, or the combination of act and clinically available drugs, such as radiotherapy, immune checkpoint blocking, anti angiogenesis therapy or myeloid regulatory drugs, may provide a more easily realized transformation path (61). Therefore, future research should strike a balance between biological complexity and producibility, scalability, safety monitoring and cost-effectiveness, especially in the field of Pediatrics and rare tumors.

Another important consideration is that act resistance should be regarded as an evolutionary and dynamic process rather than a fixed baseline feature. Under selective immune pressure, osteosarcoma lesions may evolve through antigen down-regulation or loss, epigenetic adaptation, impaired antigen presentation, metabolic reprogramming, drug-resistant subclonal amplification, or increased recruitment of inhibitory myeloid and stromal cell populations (62). These mechanisms may differ among primary tumors, lung metastases, minimal residual lesions and recurrence after treatment. Therefore, if conditions permit, longitudinal monitoring should be integrated in future ACT trials.

Serial tumor biopsies, circulating tumor DNA, circulating tumor-cell or extracellular-vesicle analyses, immune repertoire sequencing, cytokine profiling, and temporal single-cell or spatial multiomics may help distinguish antigen escape from poor trafficking, T-cell exhaustion, or TME-mediated suppression (63). This information can support adaptive treatment algorithms, including antigen re targeting, sequential or mixed ACT products, vaccine enhancement, addition of TME remodeling drugs, or early intervention in the molecular recurrence stage. From this perspective, osteosarcoma act should not be developed as a static one-time intervention, but as an adaptive treatment platform that can respond to the evolving tumor immune ecosystem.

Clinical trial design must also adapt to the biological complexity of osteosarcoma. Since many patients are children, adolescents or young adults, safety monitoring should include not only routine assessment of cytokine release syndrome and neurotoxicity, but also organ specific inflammation, pulmonary toxicity in patients with lung metastasis, long-term immune dysfunction, and potential impact on growth and development (38, 64). Dynamic biopsies, circulating tumor DNA, serial antigen assessment, cytokine profiling, and single-cell or spatial immune monitoring may help determine whether treatment failure is driven by antigen loss, poor trafficking, exhaustion, or microenvironmental suppression. Adaptive and platform trial designs may be better suited than traditional small single-arm studies to test rational combinations efficiently in this rare and biologically heterogeneous disease (65).

Ultimately, progress in osteosarcoma ACT will depend not only on maximizing cytotoxicity, but also on integrating precision biomarker selection, clinically feasible product design, longitudinal resistance monitoring, and adaptive combination strategies. Controllable, vaccine-responsive, and microenvironment-adapted cellular therapies tailored to each patient’s evolving tumor and immune landscape may provide the most realistic path toward durable benefit in this biologically heterogeneous and clinically challenging disease.
